# Analysis of clinical and genetic characteristics of Chinese children with congenital hyperinsulinemia that is spontaneously relieved

**DOI:** 10.1007/s12020-020-02585-x

**Published:** 2021-01-27

**Authors:** Zi-di Xu, Pei-pei Hui, Wei Zhang, Qiao Zeng, Lin Zhang, Min Liu, Jie Yan, Yu-jun Wu, Yan-mei Sang

**Affiliations:** 1 Department of Endocrinology, Genetics and Metabolism, Beijing Children’s Hospital, Capital Medical University, National Center for Children’s Health, 100045 Beijing, China; 2 Department of Pediatrics, Affiliated Hospital of Xuzhou Medical University, Xuzhou, 221000 China; 3 Medizinische Klinik und Poliklinik IV, Klinikum der Universität München, Ludwig-Maximilians-Universität München, Munich, 80336 Germany

**Keywords:** Congenital hyperinsulinemia, Self-spontaneous remission, Diazoxide, Octreotide, Pathogenic gene

## Abstract

**Objective:**

This study aimed to analyze the clinical and genetic characteristics of Chinese children with congenital hyperinsulinemia (CHI) that is spontaneously relieved.

**Methods:**

The patient group comprised 200 children with CHI that were treated at the Beijing Children’s Hospital from January 2006 to December 2018. The patients were divided into two groups according to their prognosis: the spontaneous remission group (*n* = 92) and the nonspontaneous remission group (*n* = 108). The clinical characteristics, pathogenic genes, diagnosis and treatment process, and follow-up data of both groups were analyzed retrospectively.

**Results:**

Of the 200 children with CHI, 92 achieved spontaneous remission. The age of spontaneous remission was between one month and nine years, and 47 of the children were relieved before the age of one year. The median age of onset was 85 days (range: 1–2825 days) in the spontaneous remission group and 2 days (range: 1–210 days) in the nonspontaneous remission group (*P* < 0.05). The mean birth weight was 3.44 ± 0.76 kg for the spontaneous remission group and 3.95 ± 0.75 kg for the nonspontaneous remission group (*P* < 0.05). Of the 92 children in the spontaneous remission group, 65 were treated with diazoxide with effective rate of 81.5% (53/65). In 12 cases in which diazoxide treatment failed, octreotide was used with an effective rate of 83.3% (10/12). Of the 108 children in the nonspontaneous remission group, 88 were treated with diazoxide with an effective rate of 43.2 % (38/88), and 29 children were treated with octreotide with an effective rate of 48.28% (14/29). Of the 30 children in the spontaneous remission group that underwent mutation analysis of CHI-related pathogenic genes, 10 children (10/30, 33.3%) carried mutations. Of the 48 children in the nonspontaneous remission group that underwent mutation analysis of CHI-related pathogenic genes, 37 children (37/48, 77.1%) were found to carry mutations. All of the differences in the indices mentioned above were statistically significant.

**Conclusions:**

The rate of spontaneous remission of CHI was significantly higher in children with late age of CHI onset, light birth weight, effective diazoxide treatment, and no common pathogenic gene mutations. Spontaneous remission was also possible for a small number of children that carried mutations in the ABCC and KCNJ11 genes and in whom diazoxide treatment failed.

## Introduction

Congenital hyperinsulinism (CHI) is a genetically heterogeneous disease in which intractable, persistent hypoglycemia is induced by excessive insulin secretion and an increase in serum insulin concentrations [1]. CHI is characterized by Intractable hypoglycemia and relative hyperinsulinemia that is not commensurate with the blood glucose level, and it is a major cause of persistent and recurrent hypoglycemia in infants and children. To date, at least 14 gene mutations have been found to be associated with CHI, corresponding to 13 genetic types [1–3]. The clinical characteristics, treatment plan, therapeutic effect, and prognosis are different for children with different types of CHI. By far the most common genetic cause of HI is inactivating mutations in ABCC8 and KCNJ11,which encode the two subunits of the pancreatic beta cell ATP-sensitive K + (KATP) channel [3]. Dominant mutations may be inherited from a parent or can arise spontaneously (i.e., de novo). Hyperinsulinism caused by dominant mutations in ABCC8 and KCNJ11may be milder and diazoxide-responsive, but some of the dominant defects may be as severe and diazoxide-unresponsive as hyperinsulinism caused by biallelic recessive KATP mutations [4]. Recent research has shown that, for some children with CHI, the hypoglycemic symptoms are gradually relieved with increasing age. In this study, 200 Chinese children with CHI were included as subjects and divided into a spontaneous remission group and a nonspontaneous remission group. The clinical and genetic characteristics of the children in each group were summarized and analyzed in order to more comprehensively understand and evaluate the prognosis of CHI and provide a scientific basis for the precise formulation of a treatment plan for CHI.

## Methods

### Subjects

A total of 200 children with CHI that were treated at the Beijing Children’s Hospital from January 2006 to December 2017 were included as the subjects, along with their parents. Out of these patients, 92 children achieved spontaneous remission during the follow-up period. All of the children included in this study were diagnosed with CHI after they were admitted to the hospital. The specific diagnostic criteria were as follows: (1) hyperinsulinemia (plasma insulin >2 uIU/mL), (2) hypolipidemia (plasma free fatty acid <1.5 mmol/L), (3) hypoketonemia (serum β-hydroxybutyric acid <2.0 mmol/L), and (4) blood glucose changes >30 mg/dL during a glucagon test with 1 mg intravenous blood [5]. The study was approved by the ethics committee of Beijing Children’s Hospital, Capital Medical University (2014-6). The parents of all participants were informed of the purpose of this study and gave their written informed consent prior to recruitment.

### Diagnosis and treatment process

After being diagnosed with CHI, 153 of the 200 children were treated with diazoxide, with an initial dose of 5 mg/(kg·d) administered orally two to three times per day. The dose was gradually increased according to the patient’s condition, with a maximum dose of 15 mg/(kg·d). When the blood glucose levels returned to normal levels, the dosage of diazoxide was gradually decreased and eventually the administration was discontinued. Diuretic hydrochlorothiazide (1–2 mg/[kg·d], administered orally two to three times per day) was used to prevent the side effects of water and sodium retention. Potassium chloride (1–2 mL of 10% potassium chloride/[kg·d], administered orally three times per day) was used to prevent hypokalemia caused by diuretics.

Of the 200 children, 41 children were treated with octreotide. The initial dose of octreotide was 5 μg/(kg·d), subcutaneously injected three to four times per day; the dose was adjusted according to the patient’s blood glucose levels. The maximum recommended dose of octreotide is generally 20 μg/(kg·d).

### Genetic analysis

#### Sample acquisition

After informed consent was obtained, genetic sequencing and analysis was conducted on 78 of the children and their parents. Three mL of venous blood was collected from the children and their parents and treated with the anticoagulant ethylenediaminetetraacetic acid. The genomic DNA of the patients and their families was extracted using the BloodGen Midi kit (CWBIO, China). All operations were carried out in strict accordance with the kit instructions.

#### Second-generation sequencing technology

A capture probe was used for the targeted capture of the exon regions of the genes associated with hypoglycemia, as identified by previous research and the Online Mendelian Inheritance in Man (OMIM) database. The chip used in the total exon capture was from the SeqCap EZ MedExome enrichment kit (Roche, Switzerland). The sequencing operation flow was standardized using the HiSeq X Ten platform (Illumina, Inc., San Diego, CA, USA).

#### First-generation sequencing (Sanger method) for validation

If the second-generation sequencing technology revealed that a child carried the mutations associated with CHI in the ABCC8, KCNJ11, GLUD1, GCK, HNF4α, HNF1α, HADH, UCP2, SLC16A1, HK1, PGM1, or CACNα1D genes, primers were designed according to the sequence of the verified loci in the gene. The DNA was amplified using the polymerase chain reaction (PCR) and sequenced with the ABI 3730 DNA analyzer (Applied Biosystems, Waltham, MA, USA). The primers used for sequencing were the same as those used in PCR amplification. The gene sequences were analyzed and compared using the DNASTAR Lasergene software (Madison, Wisconsin, USA).

#### Database analysis

Non-pathogenic mutations were excluded based on the Single-Nucleotide Polymorphism Database and the 1000 Genomes Project. The OMIM database following the American College of Medical Genetics and Genomics criteria(http://acmg.cbgc.org.cn/doku.php?id=start). The Human Gene Mutation Database, and the databases from the National Center for Biotechnology Information were used to determine whether a mutation had been previously reported.

#### Protein structure prediction software

The effects of gene mutations on protein structure were predicted using the Protein Variation Effect Analyzer software and the Sorting Intolerant from Tolerant algorithm.

### Follow-up method

After discharge, the patients were followed up for a period of one to nine years. During follow-up visits, fasting and 2-h postprandial blood glucose levels were regularly monitored; the therapeutic effect of drugs was evaluated, adverse drug reactions were monitored, and the dosage of drugs was regulated; the inducement of hypoglycemia during the treatment was recorded; and the time and age at which hypoglycemia was spontaneously relieved was recorded.

The spontaneous remission of hypoglycemia symptoms was defined as blood glucose levels maintained at ≥60 mg/dL for at least three months without treatment or after drug discontinuation [6]. The spontaneous remission was confirmed by an overnight fasting test. Using this standard for spontaneous remission, the 200 children were divided into two groups: the spontaneous remission group (*n* = 92) and the nonspontaneous remission group (*n* = 108).

### Statistical analysis

The clinical data of the spontaneous and nonspontaneous remission groups were analyzed and compared. The measurement data were expressed as the mean ± standard deviation (x ± SD). The skewed data were expressed as the median (range). SPSS 19.0 software (IBM Corp., Chicago, USA) was used for data processing. The statistical methods included the chi-square test and the analysis of variance. *P* < 0.05 was considered statistically significant for all results.

## Results

### Clinical characteristics

Of the 200 children with CHI, 117 were boys and 83 children were girls. Regarding birth weight, 78 of the children were macrosomic, 113 children had a normal birth weight, and 9 had a low-birth weight. Regarding the age of onset, 132 children developed the disease within one month of birth, 39 children developed the disease between one and six months after birth, and 29 children developed the disease more than six months after birth. Among the 200 CHI children, 142 presented with the initial symptom of seizures; 29 presented with drowsiness; 2 presented with excessive sweating; 1 presented with diarrhea; 1 presented with fatigue. The rest 25 had no symptoms. No other symptoms related to HI were observed. None of them had any risk factors for transient hyperinsulinism, such as small for dates or perinatal stress. None of them were diagnosed with such as Beckwith Weidemann Syndrome, Kabuki, Turner or Sotos.

Of the 92 children that achieved spontaneous remission, 49 were boys and 43 were girls. The children had birth weights of 1.5–6.0 kg; 24 of the children were macrosomic, 61 had a normal birth weight, and 7 had a low-birth weight. The age of onset ranged from one day to seven years and nine months: The disease developed within one month of birth in 41 children, between one and six months after birth in 23 children, and more than six months after birth in 29 children (Table 1).

**Table 1:** General clinical data of 92 CHI children that was spontaneously relieved

Clinical characteristics	Results (*n* = 92)
Male, *n* (%)	49 (53.3%)
Birth weight, kg (range)	3.44 ± 0.76 (1.5–6)
Macrosomia, *n* (%)	24 (26.1%)
Age of onset, *n* (%)	
<1 month	41 (44.6%)
1–6 months	23 (25.0%)
>6 months	28 (30.4%)
Age of spontaneous remission	
<1 month	2 (2.2%)
1–3 months	8 (8.7%)
3 months–1 year	37 (40.2%)
1–3 years	30 (32.6%)
>3 years	15 (16.3%)
Treatment of diazoxide *n* (%)	65 (70.7%)
Effective in diazoxide *n* (%)	53 (81.5%)
Gene sequencing *n* (%)	30 (32.6%)
Cases carried mutations of CHI pathogenic genes *n* (%)	10 (33.3%)

### Diagnosis and treatment effect

Treatment with diazoxide and octreotide was considered to be ineffective when the maximum dose was used for five days and the blood glucose levels of children that had fasted for more than 8–10 h could not be maintained above 70 mg/dL [7, 8].

Of the 200 children diagnosed with CHI, 153 were treated with diazoxide after they were diagnosed with CHI. The blood glucose levels of 91 of these children gradually recovered to normal levels after being treated with diazoxide, suggesting that the treatment with diazoxide was effective for these children. The blood glucose levels of the other 62 children did not significantly improve after being treated with diazoxide at the maximum recommended dose of 15 mg/(kg·d); this suggests that the treatment with diazoxide was ineffective for these children. Out of the group of 200 children, 41 children were treated with octreotide. The treatment with octreotide was effective for 24 children and ineffective for 17 children.

Of the 92 children with CHI that was spontaneously relieved, 27 cases relieved spontaneously through intensive feeding which were not treated with any medicine such as diazoxide and the other 65 were treated with diazoxide (53 were responsive to diazoxide). Octreotide was used in 12 children who were unresponsive to diazoxide. All the spontaneous remission group CHI did not underwent surgery.

Of the 108 children in the nonspontaneous remission group, 88 were treated with diazoxide(38 were responsive to diazoxide). Among the rest 50 children who were unresponsive to diazoxide, 25 were treated with Octreotide and 15 underwent surgery. Of the 15 children treated with surgery, 11 with focal lesion received partial pancreatectomy and the other four patients received total pancreatectomy. In the nonspontaneous remission group, besides the children who were treated with diazoxide, 20 were treated with (*n* = 1) or without (*n* = 19) Octreotide. None of them underwent surgery.

### Results of the genetic analysis

Of the 108 children diagnosed with CHI that was non-spontaneously relieved, 48 underwent mutation analysis of CHI-related pathogenic genes, and 37 were found to carry mutations of the known pathogenic genes (Supplemental Table 1):

(1) Thirty-one children carried mutations in the ABCC and KCNJ11 genes, of which 6 carried the compound heterozygous mutation in the ABCC8 gene, 1 carried both ABCC8 and KCNJ11 gene mutations, and 24 carried single ABCC8 or KCNJ11 gene mutations. Of the single gene mutations, 17 were paternal inherited, 2 were maternal inherited, 4 were de novo mutations., and the inheritance of the other one was unknown.

(2) Three children carried mutations in the GLUD1 gene.

(3) Three children carried other rare pathogenic mutations in the HNF4A and HADH genes.

Of the 92 children with CHI that was spontaneously relieved, 30 underwent mutation analysis of CHI-related pathogenic genes, and 10 were found to carry mutations of the known pathogenic genes (Table 2):

**Table 2:** Carrying of pathogenic gene mutations in 10 children with CHI that was spontaneously relieved

Case no	Gene mutation	Amino acid mutation	Source of mutation	Curative effect of diazoxide	Age of spontaneous remission
*ABCC8* gene					
5	c.3650G>A	p.R1217K	Maternal genetic	Effective	1 year old
10	c.4513G>C	p.D1505H	de novo	Invalid	1 year old
39	c.3455C>T	p.A1152V	Maternal genetic	Unused	5 months
42	c.1919C>T c.3586C>T	p.A640V p.Q1196X	de novo s Paternal genetic	Effective	1 year old
45	c.3000C>A c.824G>A	p.C1000X p.R275Q	Paternal genetic de novo	Effective	2 years old
49	c.4478G>A	p.R1493Q	Paternal genetic	Invalid	10 months
54	c.12Cdel	Frameshift mutations	Paternal genetic	Invalid	6 months
64	c.1484G>A	p.R495Q	Paternal genetic	Unused	1 year and 9 months
*GLUD1* gene					
92	c.1388A>G	p.N463S	de novo	Unused	9 years old
*SLC16A1* gene					
91	c.1486C>T	p.496E>K	Maternal genetic	Effective	4 years and 2 months

(1) Eight children carried mutations in the ABCC8 gene, of which two carried the compound heterozygous mutation in the ABCCA8 gene, and six carried single ABCC8 gene mutations. Of the single gene mutations, three were paternal inherited, two were maternal inherited, and one was a de novo mutation.

(2) One child carried a mutation in the GLUD1 gene.

(3) One child carried a mutation in SLC16A1 gene.

### Follow-up results for the 200 children with CHI

Of the 200 children with CHI, 92 were able to maintain normal blood glucose levels for more than three months without any further treatment and after the discontinuation of drugs; this suggests that the hypoglycemic symptoms of the patients in this group were spontaneously alleviated. The age of spontaneous remission for the 92 children was between one month and nine years, and 47 of the children were relieved before the age of one year. Table 3 compares the children in the spontaneous and nonspontaneous remission groups in terms of their general clinical characteristics and prognoses. The mutations identified in the nonspontaneous remission group are shown in Supplementary Table 1.

**Table 3:** General clinical characteristics and prognosis of children with CHI in the spontaneous remission group and the nonspontaneous remission group

	Spontaneous remission group	Non spontaneous remission group	Statistical results	*P*
Birth weight (kg)	3.44 ± 0.76	3.95 ± 0.74	F = 19.35	0.000
Age of onset (days)	85 (1–2825)	2 (1–210)	F = 18.44	0.000
Effective rate of diazoxide, n (%)	53/65 (81.54)	38/88 (43.18)	χ^2^ = 22.82	0.000
Effective rate of octreotide, n (%) Probability of carrying gene mutation, n (%)	10/12 (83.33) 10/30 (33.33)	14/29 (48.28) 37/48 (77.08)	χ^2^ = 4.30 χ^2^ = 14.76	0.038 0.000

The data is expressed as median (range) or mean ± standard deviation

## Discussion

As clinical research on CHI intensifies, more and more attention is being paid to the phenomenon of spontaneous remission in patients with CHI. A patient is characterized as having achieved spontaneous remission of CHI when normal blood glucose levels can be maintained for at least three months without medication, or medication has been stopped medication and further medical or surgical treatment is not needed. In 2011, the Banerjee team in the United Kingdom studied 101 pediatric patients with CHI and found that spontaneous remission was achieved by approximately 47.5% of the patients [5]. In a 2013 study, the Italian Sogno Valin team studied 33 children with CHI and found a spontaneous remission rate of 27.3% [9]. A study in 2016 by the Spanish Martinez team found a spontaneous remission rate of 24% for 50 children with CHI [10]. In the present study, the long-term follow-up of 200 Chinese children with CHI found a spontaneous remission rate of 46% (92/200), a result that is basically consistent with the results of the Banerjee team [6]. This suggests that spontaneous remission is an important outcome of patients with CHI, and it is necessary to study the spontaneous remission of patients with different ethnic backgrounds.

For the 92 children considered in this study that achieved spontaneous remission, the ages of onset of CHI ranged from one day to seven years and nine months, and the birth weights ranged between 1.5 and 6.0 kg. The distributions of the ages of onset and birth weights had no special characteristics. When the children in the spontaneous and nonspontaneous remission groups were compared in terms of birth weight and age of onset, it was found that the probability of spontaneous remission was significantly higher for children with a lighter birth weight and a later age of onset (*P* < 0.05, Table 3). Some children with CHI with a low gestational age may have temporary diseases [11]. In 2017, the Sakakibara A team in Japan reported that low-birth-weight infants were more likely to achieve relief of hypoglycemia [12]. However, the Banerjee team in the UK found that there was no significant difference in birth weight between the children in the spontaneous and nonspontaneous remission groups [6], which suggests that ethnic differences may be relevant to spontaneous remission.

Previous work has reported large differences in the age of spontaneous remission of children with CHI: While remission was on average achieved at 1–5 years of age [13], the latest age of remission was 12 years [14]. Working in the UK, the Banerjee team found that the earliest age of remission was six days after birth, while the latest age of remission was seven and a half years [6]. In addition, 25% of the children they studied achieved spontaneous remission within one month of birth, 48% achieved spontaneous remission within three months, and 75% achieved spontaneous remission within one year. The present study found that the age of spontaneous remission was between one month and nine years, where 51.1% (47/92) of the children achieved spontaneous remission within one year, and 75% achieved spontaneous remission within two years. Thus, the mean age of remission found in this study was slightly higher than the mean age reported in the previous literature.

Of the 200 children with CHI that took part in this study, 153 were treated with diazoxide after they were diagnosed with CHI. Out of the children treated with diazoxide, 91 achieved an effective outcome while 62 did not. Sixty-five of the 92 children in the spontaneous remission group were treated with diazoxide, and 53 achieved an effective outcome while 12 did not. The effective rate of diazoxide was 81.54% (53/65) in the spontaneous remission group, which was significantly higher than the effective rate of the nonspontaneous remission group (38/88, 43.18%; *P* < 0.05). This shows that the rate of spontaneous remission was significantly higher in patients that underwent effective diazoxide treatment, a finding that is consistent with the previous literature [6].

Forty-one of the 200 children enrolled in this study were treated with octreotide, of which 24 achieved an effective outcome while 17 did not. Octreotide was used to treat 12 of the 92 children in the spontaneous remission group, of which 10 achieved an effective outcome (10/12, 83.33%). This effective rate was significantly higher than the effective rate of the nonspontaneous remission group (14/29, 48.28%; *P* < 0.05). This shows that patients with CHI that achieved an effective outcome from octreotide treatment had a higher rate of spontaneous remission. There is no previous literature addressing the relationship between spontaneous remission and the outcome of octreotide treatment.

Of the 200 children with CHI, 78 underwent mutation analysis of CHI-related pathogenic genes, and it was found that 47 children carried mutations of these genes. Of the 31 children that did not have gene mutation, 20 achieved spontaneous remission, at a rate of 64.5% (20/31). This rate is higher than the rates reported by Martinez team in Spain (41%) and the Sogno Valin team in Italy (44%), and it is basically consistent with the rate reported by the Banerjee team in the UK (65%) [9, 10].

When the children in the spontaneous and nonspontaneous remission groups were compared, it was found that the carrier rate of gene mutation was significantly lower in the spontaneous remission group (10/30, 33.33%) than in the nonspontaneous remission group (37/48, 77.08%; *P* < 0.05). This suggests that the rate of spontaneous remission is higher in children without the mutation of CHI-related pathogenic gene, a finding that is consistent with the conclusions of the Banerjee team in the UK [9, 10].

Previous studies considered the rate of gene mutations in children with CHI that achieved spontaneous remission and in children that did not achieve spontaneous remission but were effectively treated with drugs. Approximately 15–17% of these children were found to carry mutations in one or both of the ABCC and KCNJ11 genes, while another 4–6% carried other types of gene mutations, including mutations in the GLUD1, GCK, and HNF4α genes [6, 15–17]. In the present study, genetic analysis was carried out on 30 of the 92 patients in the spontaneous remission group. According to this analysis, eight children carried ABCC8 gene mutations (8/30, 26.67%), one child carried a mutation of the GLUD1 gene (1/30, 6.7%), and one other child carried a mutation of the SLC16A1 gene (1/30, 6.7%). These proportions are higher than those reported in the previous literature; this may be due to a difference in the sample size.

The previous literature found that children with CHI that carried the maternal heterozygous mutation of the ABCC8 gene were most likely to achieve spontaneous remission, with rates of remission of 67–100% [6, 14]. In the present study, 4 of the 78 children that underwent genetic analysis were found to carry the maternal heterozygous mutation of the ABCC8 gene, and 2 of those children achieved spontaneous remission; the resulting rate of 50.0% (2/4) is consistent with the rates reported in the previous literature [6, 14]. However, a higher remission rate for individuals with maternally inherited mutation in ABCC8 compared to those with other types of mutations is probably due to the fact that ABCC8 is dominant mutation and the others may be mild mutations. Of the 20 children that were found to carry the paternal heterozygous mutation of the ABCC8 gene, 3 achieved spontaneous remission; the resulting rate of 15.0% (3/20) is slightly lower than the rate reported in the previous literature [13, 17]. Of the 8 children that were found to carry the compound heterozygous mutation in the ABCC8 gene, 2 were spontaneously alleviated, giving a remission rate of 25.0% (2/8). These results and those of previous work suggest that the detailed genetic analysis of children can be used as reference for prediction of spontaneous remission. However, adequate caution needs to be exercised when taking children off medication to ensure blood glucose levels are maintained within the safe range to avoid irreversible brain damage. Different pathogenic genes and genetic patterns are also shown to be associated with different rates of spontaneous remission. It is worth noting that the results of this study and previous work suggest that spontaneous remission may still be possible for children with ABCC and KCNJ11 gene mutations that do not achieve an effective outcome from diazoxide treatment. For these children, medical treatment must be insisted upon, and, after a longer follow-up period, it should be decided whether a pancreatectomy should be performed.

The exact mechanism of spontaneous remission in children with CHI is still not clear. Early work suggested that it is related to excessive insulin secretion, which leads to the excessive apoptosis of pancreatic β-cells. However, subsequent studies argued that spontaneous remission may be a process of functional change. 18F-L-DOPA PET/CT pancreatic scanning technology has revealed that the uptake of L-DOPA by pancreatic β-cells can still be observed in the early stage of spontaneous remission of focal pancreatic lesions. This suggests that there is no abnormality in the insulin secretion ability and that pancreatic β-cells in the lesion area lose the ability to recognize calcium ions. This mechanism may be similar to the long-term use of sulfonylureas to treat diabetic patients, which ultimately leads to a decrease in the insulin secretion function of pancreatic β-cells. Thus, the current view is that while spontaneous remission may be related to the functional shut down of insulin secretion by pancreatic β-cells, it is not necessarily accompanied by the abnormal apoptosis of pancreatic β-cells [11, 18].

In summary, the results of the present study suggest that with increasing age and need for insulin, the spontaneous remission of CHI can be achieved by some children. The rate of spontaneous remission was found to be significantly higher in children with a late age of CHI onset, a light birth weight, no mutations in CHI-related pathogenic genes, and a successful response to diazoxide treatment. In addition, there is the possibility of spontaneous remission in a small number of children that carry ABCC and KCNJ11 gene mutations and in whom diazoxide treatment has failed. Because there was found to be a large range in the age of spontaneous remission, the length of follow-up should be extended for children with CHI, and surgical treatment should be carefully selected for those children that fail to respond to medical treatment, in order to minimize the chances of surgical complications.

## Supplementary information

Supplementary Table 1
