# Supplementary material for: Analysis of clinical and genetic characteristics of Chinese children with congenital hyperinsulinemia that is spontaneously relieved
Source: Endocrine. 2021 Jan 27;72(1):116–23. doi: 10.1007/s12020-020-02585-x (PMC8087546; doi:10.1007/s12020-020-02585-x)
Supplement: Supplementary file 1 — Supplementary Table 1 [file 12020_2020_2585_MOESM1_ESM.docx]

**Supplementary Table 1: Mutations identified in the non-spontaneous remission group**

| **Case No** | **Gene mutation** | **Amino acid mutation** | **Source of mutation** | **Curative effect of diazoxide** | **Curative effect of Octreotide** | **18F-L-DOPA-PET** | **Surgery** |
| --- | --- | --- | --- | --- | --- | --- | --- |
| ***ABCC8* gene** |  |  |  |  |  |  |  |
| 99 | c.2331G>A | p.W777X | Unknown | effective | - | - | - |
| 108 | c.4703G>A | p.R1568Q | Paternal genetic | invalid | - | - | Total pancreatectomy |
| 110 | c.863G>A | p.W288X | Paternal genetic | invalid | - | - | Total pancreatectomy |
| 113 | c.4316A>G | p.L1439P | Paternal genetic | invalid | - | Focal lesion | Partial pancreatectomy |
| 121 | c.1792 C>T | p.R598X | de novo | invalid | - | Focal lesion | Partial pancreatectomy |
| 128 | c.382C>T | p.E128K | Paternal genetic | invalid | - | Focal lesion | Partial pancreatectomy |
| 129 | c.428G>A | p.W143X | Paternal genetic | invalid | - | Focal lesion | Partial pancreatectomy |
| 133 | c.106C>T | p.H36Y | Maternal genetic | - | - | - | - |
| 136 | c.47G>C | p.R16P | Paternal genetic | invalid | effective | - | - |
| 137 | c.331G>A | p.G111R | Paternal genetic | invalid | - | Focal lesion | Partial pancreatectomy |
| 139 | IVS11+2T>C | Splicing mutation | Paternal genetic | invalid | effective | - | - |
| 140 | c.1990C>T | p.G664T | Paternal genetic | - | - | Focal lesion | Partial pancreatectomy |
| 143 | c.1421A>G | p.Q474R | de novo | invalid | - | - | - |
| 144 | c.850dupG | p.A284fs | Paternal genetic | invalid | - | Focal lesion | Partial pancreatectomy |
| 145 | c.4516G>A | p.E1506K | Maternal genetic | effective | - | - | - |
| 148 | c.536A>G | p.Y179C | Maternal genetic | invalid | effective | - | - |
|  | c.3736T>C | p.W1246R | Paternal genetic |  |  |  |  |
| 149 | c.216C>A | p.N72K | Paternal genetic | effective | - | - | - |
|  | c.3733A>G | p.R1245G | Maternal genetic |  |  |  |  |
| 151 | c.1412C>T | p.A471V | Maternal genetic | invalid | effective | - | - |
|  | c.3632T>C | p.L1211P | Paternal genetic |  |  |  |  |
| 153 | c.149-2A>C (IVS1) | Splicing mutation | Paternal genetic | invalid | effective | - | - |
| 157 | c.3124_c.3126delACCinsCAGCCAGGAACTG | p.T1042Qfs*75 | Maternal genetic | invalid | effective | - | - |
|  | c.2832_c.2833inA | p.E945Rfs*25 | Paternal genetic |  |  |  |  |
| 162 | c.331G>A | p.G111R | Paternal genetic | invalid | effective | - | - |
|  | c.1792C>T | p.R598X | Maternal genetic |  |  |  |  |
| 164 | c.2800C>T | p.A934T | Paternal genetic | invalid | - | Focal lesion | Partial pancreatectomy |
| 165 | c.4414G>A | p.D1472N | Paternal genetic | invalid | effective | - | - |
|  | c.221G>A | p.R74Q | Maternal genetic |  |  |  |  |
| 167 | c.4564G>A | p.V1522M | Paternal genetic | - | - | - | - |
| 170 | c.4323delGGACCCTinsCTCAGGG | p.P1441delinsLSG | Paternal genetic | invalid | - | - | - |
| 171 | c.3127-3129dellinsCAGCCAGGAACTG | p.T1043Qfs*75 | Paternal genetic | invalid | effective | - | - |
| 173 | c.4547A>C | p.E1516A | de novo | effective | - | - | - |
| 177 | c.4463A>G | p.Q1488R | de novo | effective | - | - | - |
| 185 | c.1541G>A | p.W514X | Paternal genetic | effective | - | - | - |
| ***ABCC8* gene and *KCNJ11* gene** | |  |  |  |  |  |  |
| 138 | ABCC8  c.1775A>C | p.L592R | Maternal genetic | effective | - | - | - |
|  | KCNJ11 c.374T>C | p.I125T | Unknown |  |  |  |  |
| ***KCNJ11* gene** |  |  |  |  |  |  |  |
| 193 | c. 703C>G | p. Q235E | Paternal genetic | invalid | - | - | - |
| ***GLUD1* gene** |  |  |  |  |  |  |  |
| 141 | c.1388A>G | p.N463I | de novo | effective | - | - | - |
| 183 | c.1495C>A | p.G499C | de novo | effective | - | - | - |
| 194 | c.978G>A | p.R269H | Paternal genetic | effective | - | - | - |
| ***HNF4α* gene** |  |  |  |  |  |  |  |
| 180 | c.481C>T | p.P161S | Maternal genetic | invalid | - | Focal lesion | Partial pancreatectomy |
| 181 | c.157T>C | p.C53R | de novo | effective | - | - | - |
| ***HADH* gene** |  |  |  |  |  |  |  |
| 184 | c.419+1G>A (IVS3) | Splicing mutation | Paternal genetic | effective | - | - | - |
|  | c.547-1G>C (IVS4) | Splicing mutation | Maternal genetic |  |  |  |  |
